# Regulation of the Interfaces Between Argyrodite Solid Electrolytes and Lithium Metal Anode

**DOI:** 10.3389/fchem.2022.837978

**Published:** 2022-02-01

**Authors:** Bo Pang, Yongping Gan, Yang Xia, Hui Huang, Xinping He, Wenkui Zhang

**Affiliations:** College of Materials Science and Engineering, Zhejiang University of Technology, Hangzhou, China

**Keywords:** argyrodite solid electrolyte, lithium metal anode, lithium dendrites, interface reaction, all-solid-state lithium batteries

## Abstract

Lithium-ion batteries (LIBs) are widely used in portable electronic devices, electric vehicles and large scale energy storage, due to their considerable energy density, low cost and long cycle life. However, traditional liquid batteries suffer from safety problems such as leakage, thermal runaway and even explosion. Part of the issues are caused by lithium dendrites puncturing the liquid electrolyte during cycling. In order to achieve the objective of higher safety and energy density, a rigid solid-state electrolyte (SSE) is proposed instead of liquid electrolyte (LE). Thereinto, sulfide SSEs have received of the most attention due to their high ionic conductivity. Among all the sulfide SSEs, argyrodite SSEs are considered to be one of the most promising solid-state electrolytes due to their high ionic conductivity, high thermal stability and good processablity. On the other hand, lithium metal is an ideal material for anode because of its high specific energy, low potential and large storage capacity. However, interfacial problems between argyrodite SSEs and the anode (interfacial reactions, lithium dendrites, etc.) are considered to be important factors affecting their availability. In this mini review, we summarize the behavior, properties and problems arising at the interface between argyrodite SSEs and anode. Strategies to solve interface problems and stabilize interfaces in recent years are also discussed. Finally, a brief outlook about argyrodite SSEs is presented.

## Introduction

With the large-scale use of electrification and the development of energy storage science and technology, there is an urgent demand for a new generation of energy storage materials with high energy density, high safety and long cycle life. Lithium metal has the advantages of high theoretical capacity, low potential and large reserves, has been attracting much attention. The advent of the liquid lithium-ion battery (LIB) has increased the utilization and storage of electrical energy as a result of its high energy density and long cycle life. However, traditional LIBs have safety issues such as leakage and thermal runaway caused by lithium dendrites piercing the liquid electrolyte. In order to solve the safety problem and improve the energy density of the battery, solid-state electrolyte (SSE) is proposed and researched intensively. Inorganic solid electrolytes can be divided into sulfide solid electrolytes, oxide solid electrolytes, halide solid electrolytes, and ect (Tang et al., 2018).

The sulfide solid electrolyte structure is derived from oxide by replacing the oxygen element by sulfur, which expands its original ion radius and thus making the lithium ion transport channel larger (Minami et al., 2000). Since the discovery of binary sulfide solid electrolyte systems Li_2_S-GeS_5_, Li_2_S-P_2_S_5_, Li_2_S-B_2_S_3_ has been reported, the most studied glassy sulfide solid electrolyte is the Li_2_S-P_2_S_5_ system (Mercier et al., 1981; Zhang and Kennedy, 1990; Kanno and Murayama, 2001; Zhang et al., 2019a; Zhang et al., 2020a). Glassy sulfide SSEs exhibits higher ionic conductivity than other sulfide SSEs because of the characteristics of intimate particle contact during the extrusion molding process and the eliminated grain boundary resistances (Janek and Zeier, 2016). In the xLi_2_S-(100-x)P_2_S_5_ system, Li_3_PS_4_ (75Li_2_S-25P_2_S_5_) (Tatsumisago et al., 2002), Li_7_PS_6_ (88 Li_2_S-P_2_S_5_) (Kong et al., 2010), Li_7_P_3_S_11_ (70Li_2_S-30P_2_S_5_) (Yamane et al., 2007), and Li_4_P_2_S_6_ (67Li_2_S-33P_2_S_5_) (Mercier et al., 1982), exhibit relatively high ionic conductivity. In 1976, Goodenough’s team (Goodenough et al., 1976) synthesized NASICON-type oxide solid-state electrolytes by high-temperature solid-phase development. On this basis, the researchers used lithium ions to replace sodium ions and introduced sulfur to synthesize Thio-lithium superionic conductor (Thio-LISICON). Among Thio-LISICONs, Kanno’s team (Kamaya et al., 2011) reported Li_10_GeP_2_S_12_ (LGPS) with the highest room temperature conductivity (12 mS cm^−1^). The high conductivity of Li_10_GeP_2_S_12_ can be attributed to the fact that the electrolyte has a two-dimensional lithium-ion transport channel in the ab plane of the cell and a one-dimensional fast lithium-ion transport channel in the c axis. Besides, a series of Thio-LISICON of Li_10±1_MP_2_S_12_ (M = Si, Ge, Sn, Ga, Sb or P) was also developed. Li_10_SnP_2_S_12_, as an isomer of LGPS, has gained widespread attention because of the high reserves of Sn elements and low cost (Bron et al., 2013), while ensuring its room temperature conductivity of 4 × 10^–3^ S cm^−1^. In Table 1 we have listed several sulfide solid electrolytes and described their properties.

**TABLE 1:** Summary of the performance of sulphide solid electrolytes.

Sulfide solid electrolytes	Material type	Conductivity (S cm^−1^)	Reference
Li_7_P_3_S_11_	Glass–ceramic	5.2 × 10^−3^	Minami et al. (2011)
Li_7_P_2.9_S_10.85_Mo_0.01_	Crystal	4.8 × 10^–3^	Xu et al. (2017)
Li_10_GeP_2_S_12_	Crystal	1.2 × 10^–2^	Kamaya et al. (2011)
Li_10_SnP_2_S_12_	Crystal	4 × 10^–3^	Bron et al. (2013)
Li_6_PS_5_Cl	Crystal	1.33 × 10^–3^	Boulineau et al. (2012)
Li_6_PS_5_Br	Crystal	2.58 × 10^–3^	Yu et al. (2019)
Li_6_PS_5_C_l0.25_Br_0.75_	Crystal	3.4 × 10^–3^	Zhou et al. (2019b)
Li_6_PS_5_I (with excess Li_2_S)	Crystal	1.5 × 10^–5^	Ge et al. (2018)

**FIGURE 1:**
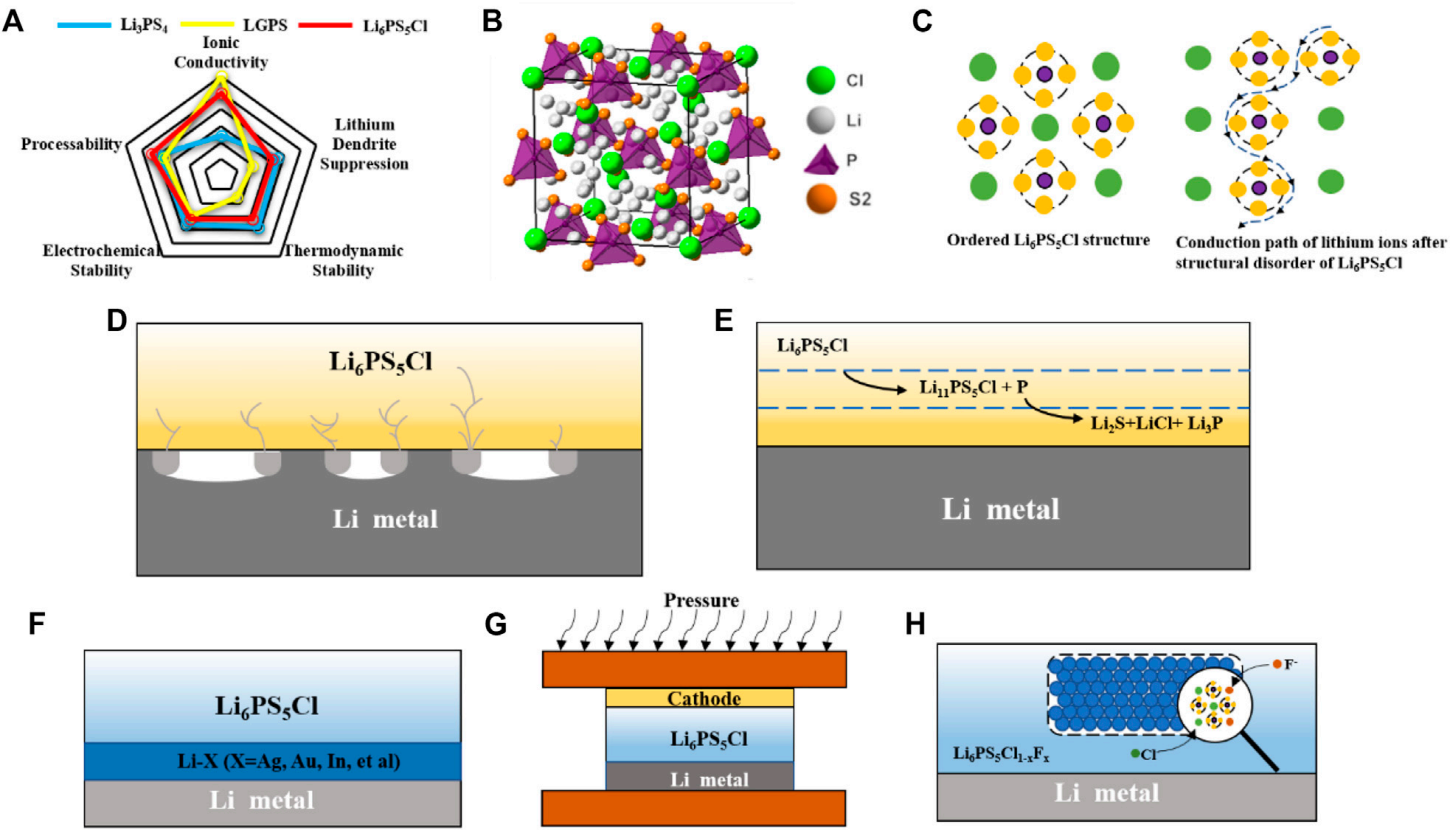
**(A)** Crystal structures of argyrodite sulfide SSEs. **(B)** Conduction paths of lithium ions in argyrodite SSEs with different degrees of disorder. **(C)** Comparison of performance of three typical sulfide SSEs. **(D)** Schematic of lithium dendrite growth. **(E)** Schematic of interface reaction. **(F)** Schematic of alloy negative electrode. **(G)** Pressurization during battery cycling. **(H)** Schematic of doping using F instead of Cl element.

Although LGPS has an ionic conductivity that compares favorably with that of liquid, but is limited in practical applications by side reactions with the lithium metal anode and narrow electrochemical window. While the LPS system is compatible with mainstream cathode and anode, its low ionic conductivity limits its development prospects. In recent years, argyrodite SSEs have received a lot of attention from researchers due to their high ionic conductivity and relatively stable crystal structure (Zhang et al., 2018). Compared with LGPS and LPS electrolytes, argyrodite SSEs has both high ionic conductivity and good interfacial compatibility (Figure 1A). However, argyrodite SSEs still has many aspects that need to been improved. Problems such as interfacial reactions and disordered growth of lithium dendrites can still occur in argyrodite SSEs and Li-metal anode. To implement argyrodite SSEs for practical applications, the problem at the interface must be solved. In this review, various problems at the interface between argyrodite SSEs and Li anode are comprehensively described, and the research strategies proposed to improve the interface in recent years are summarized. Finally, strategies for these interfacial problems and outlooks are prospected.

Inspired by the structure of argyrodite Ag_8_GeS_6_, Deiseroth’s team (Deiseroth et al., 2008) has developed a Li_6_PS_5_X (X = Cl, Br or I) sulfide solid-state electrolyte. Structural studies have shown that Li_6_PS_5_X represents a series of argyrodites whose chemical formulae are based on the known substitution of a halogen atom for an Ag or Cu atom. The structure of Li_6_PS_5_X is a cubic crystal structure in which the S^2−^ anion is located in the tetrahedral gap at 16e, P at 4b, and the halogen group elements are located at the apex and face center of the cubic crystal structure. Besides, the structure has four individual S atoms at the 4C position surrounded by 18 Li^+^ (Figure 1B). These Li^+^ occupy these positions, and the other half are empty spaces through which Li+ may migrate in the structure (Kraft et al., 2017). In addition to the lithium-ion vacancies, the introduction of different halogen elements also has a great influence on the lithium-ion diffusion in argyrodite SSEs. The difference in conductivity among Li_6_PS_5_Cl (1.9 × 10^–3^ S cm^−1^), Li_6_PS_5_Br (6.8 × 10^–4^ S cm^−1^), and Li_6_PS_5_I (4.6 × 10^–7^ S cm^−1^) depends on the degree of S-X disorder. Since the ionic radii of halogens Cl^−^, Br^−^ are similar to S^2−^, the introduction of Cl^−^, Br^−^ has an obvious site-specific disorder. While I^−^ exhibits an ordered distribution with S^2−^ because of its larger ionic radius, which leads to the room temperature conductivity of Li_6_PS_5_I is not as high as the other. Baktash et al. (2020) found that increasing the S-Cl disorder can substantially increase the Li_6_PS_5_Cl ionic conductivity. Nazar et al. (Zhou et al., 2019a) further found that the activation energy barrier to reduce the mobility of lithium ions is the key to the disordered distribution of Li^+^. In addition, their work shows that the use of other elements (Ge, Si, etc.) in partial substitution of phosphorus in addition to halogen atoms can also cause Li^+^ site disorder. From the above research work, it can be found that anion disorder and the resulting lithium disorder is the key to achieve rapid diffusion of lithium ions (Figure 1C).

## Interfaces Between Argyrodite Solid-State Electrolytes and Lithium-Metal Anode

Although argyrodite SSEs have high ionic conductivity and relatively good stability at the interface with the anode. However, compared with conventional liquid electrolytes, argyrodite SSEs have ineligible interfacial problems with the anode. Interfacial problems are mainly divided into two aspects as following: one is the growth of lithium dendrites, and the other one is the interfacial reactions. In conventional liquid batteries, the lithium metal is well infiltrated by the liquid electrolyte, which helps to induce relatively smooth lithium deposition. The argyrodite SSEs will have a critical current density in the process of cycling with the lithium anode. When the current density is exceeded the critical current density during plating or stripping, dendritic lithium deposition will form and cause short circuit. When the current density of the stripped lithium exceeds its own rate of replenishment, the lithium metal forms voids at the interface and aggravate the uneven deposition of lithium. (Koerver et al., 2017; Manalastas et al., 2019) Bruce et al. (Kasemchainan et al., 2019) found that these voids will only be partially eliminated in the following cycles, and the remainder will gradually accumulate as the cycles increase. The accumulation of these voids reduces the contact area at the interface and increases the local current density, which eventually leads to the generation of lithium dendrites (Figure 1D). In further work, they also found a close relationship between the magnitude of the critical current density leading to lithium dendrite generation and pressure. When the applied pressure is greater than 0.81 MPa, the lithium metal is deformed plastically, and creep rather than diffusion of the lithium metal is the main mechanism for lithium transport to the interface. Ning et al. (2021) proposed that lithium dendrites are inextricably linked to cracks of SSEs generated during cycling. Studies using *in situ* X-ray computed tomography revealed that small cracks appear first at the edges of the electrolyte during lithium deposition. The current density is enhanced at the edges of these cracks, inducing the deposition of lithium (Tang et al., 2009). With more and more lithium depositions at the cracks, the original cracks show dendritic expansion. The formation of lithium dendrites has a great relationship with the local current density, so that the control of the local current density can inhibit the generation of lithium dendrites.

In addition to the lithium dendrite issue, the interfacial reactions induced by the thermodynamic instability between argyrodite SSEs and anode are also worth of attention. Juergen et al.(Wenzel et al., 2018) dopted X-ray photoelectron spectroscopy to confirm that Li_6_PS_5_X will decompose at interface due to the strong reduction of lithium. Some of the Li_6_PS_5_X decomposed on the surface of lithium metal to form solid electrolyte interphase (SEI) with the ingredient of decomposition products such as Li_3_P, Li_2_S and LiX (Figure 1E). The SEI leads to an increase in interfacial resistance and hinders the conduction of lithium ion. Further study by Wagemaker et al. (Schwietert et al., 2020) revealed that the thermodynamic decomposition reaction of argyrodite SSEs with the anode side is not instantaneous. Taking argyrodite Li_6_PS_5_Cl as an example, it was first reduced to the unstable Li_11_PS_5_Cl, and then Li_11_PS_5_Cl was further reduced to Li_2_S, LiCl and Li_3_P. In this process, the generated intermediate phases such as S, Li_2_S and LiCl not only produce a large volume expansion, but also leading to the destabilization of the kinetics. The continued decomposition of argyrodite SSEs at the interface as the cycle proceeds is the main reason for the increase in interfacial resistance. Our group (Zheng et al., 2021) used *in situ* Raman energy spectroscopy and *in situ* electrochemical impedance spectroscopy to find “self-healing” effect of Li_6_PS_5_Cl after decomposition. The Li_6_PS_5_Cl self-healing mechanism is mainly attributed to the reversible redox reaction that can be achieved by Li_6_PS_5_Cl and the consumption of the deposited lithium metal during the redox reaction. The discovery of the self-healing mechanism of argyrodite SSEs provides a new idea and theoretical basis to tackle interface problems.

## Strategies for the Interfacial Issues

Considering the complex interfacial problem between argyrodite SSEs and Li-metal anode, strategies were proposed from different perspectives. The researchers firstly proposed using an alloy to react with the lithium anode or building a suitable 3D collector to solve the interfacial problem of lithium dendrite. Secondly, the argyrodite SSEs are doped with different elements to artificially construct SEI during the contact with the lithium anode, or to make the argyrodite SSEs flexible or three-dimensional structure to suppress the interfacial reaction.

### Alloyed Anode and 3D Current Collectors

Lithium metal has been a popular anode material because of its high specific capacity and the lowest redox potential. However, in practical applications, the generation of lithium dendrites at the interface has hindered its practical application and development. The method of synthesizing alloy anode by lithium and alloy can effectively regulate the growth of lithium dendrites and induce the uniform deposition of lithium during the cycling process. By studying the nucleation mechanism of lithium on different metals, Cui et al. (Yan et al., 2016) found that lithium is deposited on different metals with different overpotentials. The selection of different alloy substrates can be used to tune the lithium metal deposition. Wang et al. (Zhang et al., 2021) concluded from the binary phase diagram to explain that lithium exhibits good structural stability during alloying with silver and can form lithium-silver alloys in different ratios. After cycling to lithium stripping, the de-lithiumed porous silver particles can be used as a carrier material to induce the next lithium deposition. While gold and lithium form a lithium-saturated material with limited ability to retain lithium and cannot regulate the deposition of excess lithium, leading to structural instability. Based on the above study, silver is an excellent alloy conductor, which can form various types of lithium-silver solid solutions with lithium continuously and regulating the deposition of lithium. Therefore, Kim et al. (Choi et al., 2022) proposed a Ag-Li alloy anode could be made in large quantities by roll pressing (Figure 1F). In contrast to previous reports, *in situ* formation of silver-rich silver-lithium intermetallic compounds can induce uniform deposition of lithium after simple roll aging. The Ag-Li spacer can play a role in inducing a uniform deposition of lithium during the cycling process, rather than concentrating on one spot, thus maintaining a more stable SE/Ag-Li interface. By adding a layer of Ag-Li between the lithium anode and Li_6_PS_5_Cl, normal cycling at very high current density (12C) was achieved and the growth of lithium dendrites was well suppressed. In addition to the use of alloy anode, lithium-free graphite can also inhibit the growth of lithium dendrites due to its inherent 3D structure. Han et al. (Lee et al., 2020) designed an Ag-C anode that achieves 1000 cycles at high energy density with a Coulombic efficiency of more than 99.8%. Using Ag-C composite anode, the problems of low energy density and cycle life of conventional argyrodite batteries are well solved by the regulation of lithium dendrites of Ag and the 3D structure of graphite to accommodate the volume expansion. Li et al. (Ye and Li, 2021) constructed a sandwich structure of LPSCl-LGPS-LPSCl to overcome the instability between LGPS and Li. The “expansion screw effect” of the LGPS decomposition is used to fill the empty space created by the decomposition of the LPSCL. This property can inhibit the growth of lithium dendrites.

In addition to the widespread use of silver and lithium to form lithium-silver alloys to regulate the growth of lithium dendrites, other alloying elements also have their advantages in regulating lithium dendrites. Zhang et al. (Luo et al., 2021) took advantage of the inherent good deformability of the Li-In alloy to assemble the cell under high pressure to make a tighter fit between the electrolyte and the negative electrode of the alloy (Figure 1H). The close contact between the electrolyte and the anode eliminates the voids at the interface and suppresses the generation of lithium dendrites. Hayashi et al. (Kato et al., 2018) took advantage of the fact that Au can form a lithium-gold alloy with lithium to add a layer of gold foil between the electrolyte and the lithium anode, increasing the sites for lithium deposition, while the resulting lithium-gold alloy can fill the gap formed after the lithium is stripped well. Due to the greater binding energy with lithium than the electrolyte, alloying elements will preferentially combine with lithium metal to form Li-alloy anode, thereby leading to uniform deposition of lithium and avoiding lithium dendrite generation.

### Modification of the Argyrodite Solid-State Electrolytes

In addition to the formation of lithium dendrites due to the inhomogeneous deposition of lithium, another serious interfacial problem between lithium metal anode and argyrodite SSEs is the interfacial reaction (Xu et al., 2018a; Zhang et al., 2019b). Due to the high reduction of lithium metal anode, argyrodite SSEs easily reduced by lithium metal at the interface and decomposed into intermediate phases such as Li_2_S, Li_3_P. The passivation layer composed of these intermediate phases will increase the interfacial impedance as the battery cycle progresses, resulting in degradation of the battery performance. It has been previously reported (Xu et al., 2018b)^,^ (Fan et al., 2018) that pretreatment of lithium metal surfaces with chemicals containing I or F can effectively reduce interfacial resistance and inhibit interfacial reactions (Zhang et al., 2020b). In order to improve the stability of the lithium metal anode/argyrodite interface, Sun et al. (Zhao et al., 2020) proposed the fluorination of Li_6_LP_5_Cl. The fluorinated LPSC_l0.3_F_0.7_ electrolyte was synthesized by introducing the element F into the Li_6_LP_5_Cl electrolyte using a solid-state synthesis method. During the cycling process, a dense and well-conducting LiF is *in situ* generated at the interface between LPSC_l0.3_F_0.7_ and lithium metal (Figure 1H). The sheet-like LiF prevents the contact between the electrolyte and the lithium metal, thus inhibiting the interfacial reactions. Tu et al. (Liu et al., 2021) synthesized LPSNCl by solid-phase method using partial substitution of N atoms for S atoms and discovered that the tetrahedral position occupied by N atoms in the crystal could make the crystal structure more stable. During the cycling process, the electrolyte generates a LiN-rich SEI layer *in situ* on the surface of the lithium anode, successfully curbing the continual occurrence of interfacial reactions. Apart from doping of other elements to induce the *in situ* generation of SEI to suppress the interfacial reaction, the construction of three-dimensional and flexible electrolytes can also contain the interfacial reaction. Zhu et al. (Cao et al., 2021) synthesized flexible and highly ion-conductive electrolyte films using ethylcellulose and LPSCl. The electrolyte film can fully contact with the lithium metal anode, inhibit the growth of lithium dendrites under high pressure, and its own excellent thermal stability can inhibit the decomposition of the electrolyte film during cycling. Cui et al. (Wang et al., 2021) synthesized a three-dimensional porous LPSCl skeleton using SeS_2_ as pore-forming agent, which reduced the problem of poor solid-solid contact between electrolyte and anode by means of *in situ* polymerization and greatly reduced the interfacial resistance.

## Summary and Outlook

Argyrodite SSEs are attracting more and more attention because of their high ionic conductivity and their self-healing mechanism when reacting with lithium metal anode. Nevertheless, the complex interface problems between argyrodite SSEs and lithium metal anode limit their practical use. Growth of lithium dendrites due to excessive local current density from voids created during lithium stripping/deposition when matched with argyrodite SSEs. Because of the strong reduction of lithium, the increasing interfacial resistance due to the continuous decomposition of argyrodite SSEs at the Li-metal anode interface is another interface problem which limits the practical application of argyrodite SSEs. In response to these obvious interface problems, a number of solutions have been proposed. The construction of the alloy anode can effectively guide the uniform deposition of lithium, thus reducing the growth of lithium dendrites due to uneven deposition during lithium stripping. Elemental doping of argyrodite SSEs allows the electrolyte to generate a dense SEI film *in situ* with the lithium metal anode during cycling, which can inhibit the continual occurrence of interfacial reactions. Although many interface issues still need to be resolved, the future of argyrodite batteries is still worthy of anticipation. We present our outlook on interface issues from the following perspectives.

1) Mechanism of interfacial lithium deposition need to be furtherly explored. Although a large amount of work has been done to summarize the mechanism of lithium ion transport at the interface. The issues such as the preferred sites for lithium deposition during deposition, the connection between the voids created by exfoliation and the deposition sites, still lack in-depth studies. We expect to use more three-dimensional and clearer characterization tools to characterize the whole process of lithium dendrite growth. Combining these characterization tools with the study of interfacial mechanisms will lead to more specific solutions.
2) Development of novel argyrodite SSEs. For argyrodite SSEs, the behavior of spontaneous decomposition during contact with lithium metal limits their use. The application of various elemental covalently generates a dense SEI layer *in situ* to suppress the interfacial reaction while maintaining the ionic conductivity.
